# Supplementary material for: Miniaturized 3D bone marrow tissue model to assess response to Thrombopoietin-receptor agonists in patients
Source: eLife. 2021 Jun 1;10:e58775. doi: 10.7554/eLife.58775 (PMC8169123; doi:10.7554/eLife.58775)
Supplement: Transparent reporting form [file elife-58775-transrepform.docx]

***eLife’s* transparent reporting form**

We encourage authors to provide detailed information *within their submission* to facilitate the interpretation and replication of experiments. Authors can upload supporting documentation to indicate the use of appropriate reporting guidelines for health-related research (see [EQUATOR Network](http://www.equator-network.org/%20)), life science research (see the [BioSharing Information Resource](https://biosharing.org/)), or the [ARRIVE guidelines](http://www.plosbiology.org/article/info:doi/10.1371/journal.pbio.1000412) for reporting work involving animal research. Where applicable, authors should refer to any relevant reporting standards documents in this form.

If you have any questions, please consult our Journal Policies and/or contact us: [editorial@elifesciences.org](mailto:editorial@elifesciences.org).

**Sample-size estimation**

- You should state whether an appropriate sample size was computed when the study was being designed
- You should state the statistical method of sample size computation and any required assumptions
- If no explicit power analysis was used, you should describe how you decided what sample (replicate) size (number) to use

Please outline where this information can be found within the submission (e.g., sections or figure legends), or explain why this information doesn’t apply to your submission:

Sample size was not estimated before-hand because the phenotypic outcome of the intervention (treatment with Thrombopoietin *vs* Thrombopoietin plus Eltrombopag) was completely unknown, so we could not estimate an expected variation of the parameters analyzed to determine a sample size. Further, inherited thrombocytopenias are rare diseases caused by more than 30 different described genetic mutations, with nearly half of the patients affected by still unknown mutations. For all these reasons, we have kept the samples at the maximum to achieve statistical and biological significance. This included the recruitment of patients harboring mutations in two of the most frequent affected genes, *ANRKD26* and *MYH9*. Specifically, we have processed 24 samples from 20 different patients, each divided into the two experimental conditions (total of 48 investigated samples). 13 patients had previously received a short-term course of Eltrombopag either within a phase 2 clinical trial (Zaninetti et al., 2020) (n = 11) or in preparation for elective surgery (Zaninetti et al., 2019) (n = 2). For 4 patients, the analysis was performed on two different occasions. Heathy controls were routinely cultured in parallel to diseased samples.

Sample size is reported in ‘*Material and Methods*’ (*Patients* paragraph) and in Table 1.

**Replicates**

- You should report how often each experiment was performed
- You should include a definition of biological versus technical replication
- The data obtained should be provided and sufficient information should be provided to indicate the number of independent biological and/or technical replicates
- If you encountered any outliers, you should describe how these were handled
- Criteria for exclusion/inclusion of data should be clearly stated
- High-throughput sequence data should be uploaded before submission, with a private link for reviewers provided (these are available from both GEO and ArrayExpress)

Please outline where this information can be found within the submission (e.g., sections or figure legends), or explain why this information doesn’t apply to your submission:

A biological replicate is intended as the analysis of multiple samples from different patients or different induced pluripotent stem cell (iPSC) clones in the same experimental condition. Data are clustered based on the genotype (Healthy controls-Wyle Type, *ANKRD26-*RT*, MYH9*-RD) and the treatment allocation (Thrombopoietin vs Thrombopoietin plus Eltrombopag). This applies to all the experiments performed with megakaryocytes from human blood samples and iPSC clones. For 4 patients, the analysis was performed two times. These analyses are intended as biological replicates as they were performed in two different independent occasions. The numbers of biological replicates are indicated in the *Figure Legends*.

A technical replicate is intended as repeated analysis performed on the same sample at the same time. This applies to the experiments of morphologic and genomic characterization performed on iPSC clones. The numbers of technical replicates are indicated in the *Figure Legends*.

All data was included, none was excluded.

The size number of each experiments in reported in all the *Figure Legends*. A statement related to the minimum number of replicates for the statistical analysis is reported in ‘*Material and Methods*’ (*Statistics* paragraph).

**Statistical reporting**

- Statistical analysis methods should be described and justified
- Raw data should be presented in figures whenever informative to do so (typically when N per group is less than 10)
- For each experiment, you should identify the statistical tests used, exact values of N, definitions of center, methods of multiple test correction, and dispersion and precision measures (e.g., mean, median, SD, SEM, confidence intervals; and, for the major substantive results, a measure of effect size (e.g., Pearson's r, Cohen's d)
- Report exact p-values wherever possible alongside the summary statistics and 95% confidence intervals. These should be reported for all key questions and not only when the p-value is less than 0.05.

Please outline where this information can be found within the submission (e.g., sections or figure legends), or explain why this information doesn’t apply to your submission:

Values were expressed as mean plus or minus the standard deviation (mean±SD) or mean plus or minus the standard error of the mean (mean±SEM). A two-tailed paired t-test was performed for statistical analysis of data from samples tested in parallel under different experimental conditions. A two-tailed unpaired t-test was performed for statistical analysis of data from different samples. Statistical analysis was performed with GraphPad Software. A p-value of less than 0.01 or 0.05 was considered statistically significant. All experiments were independently replicated at least three times.

(For large datasets, or papers with a very large number of statistical tests, you may upload a single table file with tests, Ns, etc., with reference to sections in the manuscript.)

**Group allocation**

- Indicate how samples were allocated into experimental groups (in the case of clinical studies, please specify allocation to treatment method); if randomization was used, please also state if restricted randomization was applied
- Indicate if masking was used during group allocation, data collection and/or data analysis

Please outline where this information can be found within the submission (e.g., sections or figure legends), or explain why this information doesn’t apply to your submission:

All samples from inherited thrombocytopenic patients and iPSC clones were split and allocated to both experimental groups (Thrombopoietin *vs* Thrombopoietin plus Eltrombopag). No randomization was applied.

Results related to platelet response to Eltrombopag during *in vivo* treatment were masked to the laboratory until collection and analysis of data from *ex vivo* treatment of samples from the same patient.

**Additional data files (“source data”)**

- We encourage you to upload relevant additional data files, such as numerical data that are represented as a graph in a figure, or as a summary table
- Where provided, these should be in the most useful format, and they can be uploaded as “Source data” files linked to a main figure or table
- Include model definition files including the full list of parameters used
- Include code used for data analysis (e.g., R, MatLab)
- Avoid stating that data files are “available upon request”

Please indicate the figures or tables for which source data files have been provided:

All the information related to the experimental data is available in the figures, tables and/or as supplementary files containing raw data with reference to main figures.
